# Whole-genome duplication increases tumor cell sensitivity to MPS1 inhibition

**DOI:** 10.18632/oncotarget.6432

**Published:** 2015-11-30

**Authors:** Mohamed Jemaà, Gwenola Manic, Gwendaline Lledo, Delphine Lissa, Christelle Reynes, Nathalie Morin, Frédéric Chibon, Antonella Sistigu, Maria Castedo, Ilio Vitale, Guido Kroemer, Ariane Abrieu

**Affiliations:** ^1^ CRBM, CNRS UMR5237, Université de Montpellier, Montpellier, France; ^2^ Regina Elena National Cancer Institute, Rome, Italy; ^3^ Université Paris-Sud/Paris XI, Le Kremlin-Bicêtre, France; ^4^ INSERM, UMRS1138, Paris, France; ^5^ Equipe 11 Labelisée par la Ligue Nationale Contre le Cancer, Centre de Recherche des Cordeliers, Paris, France; ^6^ Gustave Roussy Cancer Campus, Villejuif, France; ^7^ EA 2415, Laboratoire de Biostatistique, d'Epidémiologie et de Recherche Clinique, Université de Montpellier, Montpellier, France; ^8^ Department of Biopathology, Institut Bergonié, Comprehensive Cancer Centre, Bordeaux, France; ^9^ INSERM U916, Bordeaux, France; ^10^ Department of Biology, University of Rome “Tor Vergata”, Rome, Italy; ^11^ Université Pierre et Marie Curie/Paris VI, Paris, France; ^12^ Pôle de Biologie, Hôpital Européen Georges Pompidou, AP-HP, Paris, France; ^13^ Metabolomics and Cell Biology Platforms, Gustave Roussy Cancer Campus, Villejuif, France

**Keywords:** AZ 3146, mitotic spindle, polyploidy, regulated cell death, reversine

## Abstract

Several lines of evidence indicate that whole-genome duplication resulting in tetraploidy facilitates carcinogenesis by providing an intermediate and metastable state more prone to generate oncogenic aneuploidy. Here, we report a novel strategy to preferentially kill tetraploid cells based on the abrogation of the spindle assembly checkpoint (SAC) via the targeting of TTK protein kinase (better known as monopolar spindle 1, MPS1). The pharmacological inhibition as well as the knockdown of MPS1 kills more efficiently tetraploid cells than their diploid counterparts. By using time-lapse videomicroscopy, we show that tetraploid cells do not survive the aborted mitosis due to SAC abrogation upon MPS1 depletion. On the contrary diploid cells are able to survive up to at least two more cell cycles upon the same treatment. This effect might reflect the enhanced difficulty of cells with whole-genome doubling to tolerate a further increase in ploidy and/or an elevated level of chromosome instability in the absence of SAC functions. We further show that MPS1-inhibited tetraploid cells promote mitotic catastrophe executed by the intrinsic pathway of apoptosis, as indicated by the loss of mitochondrial potential, the release of the pro-apoptotic cytochrome *c* from mitochondria, and the activation of caspases. Altogether, our results suggest that MPS1 inhibition could be used as a therapeutic strategy for targeting tetraploid cancer cells.

## INTRODUCTION

Aneuploidy, the condition of having an imbalanced copy number of chromosomes (DNA content ≠ x*n*, where *n* stands for the haploid chromosome set and *x* ≥ 1), and chromosome instability (CIN), a type of genomic instability in which cells display an elevated rate of whole-chromosome mis-segregations (∼1 per 5 cell divisions) and thus frequently change their karyotype [1], are widespread in human tumors [2–5]. Along with this, variations of chromosome number have been linked to cancer progression and aggressiveness [4, 5], as well as therapeutic resistance [6, 7] and poor patient prognosis [8, 9], although their precise impact in tumorigenesis is still debated (for recent reviews refer to [10]).

One prominent mechanism accounting for the generation of aneuploidy in cancer involves a preliminary and unscheduled passage to a tetraploid intermediate (DNA content = 4*n*) [11–13]. Tetraploid cells are generated by a variation of the canonical G_1_-S-G_2_-M cell cycle, such as skipped (*i.e*., endocycling/endoreplication) or aborted (*e.g*., endomitosis or cytokinesis failure) mitoses, or by cell membrane fusion [14–18]. According to the current hypotheses, illicitly generated tetraploids are less likely to stably maintain their karyotype across consecutive generations due to intrinsic defects in the machineries involved in DNA replication, DNA repair and/or chromosome segregation [19–21]. These defects might increase the level of CIN, in turn resulting in the generation of aneuploid cells [14, 19, 22]. The evidence supporting this two-step cascade in tumor development includes (1) indirect observations, such as the elevated incidence of tetraploid cells in early stages of tumors and in pre-neoplastic lesions, where tetraploidy appearance often precedes the acquisition of CIN and the subsequent development of aneuploidy [23–26]; (2) mathematical models, which indicate that the gradual loss of chromosomes from a tetraploid intermediate rather than chromosome(s) loss or gain during mitotic divisions of diploid cells accounts for the near-triploid/tetraploid content frequently found in solid tumors [2, 27]; (3) computational studies, inferring that around 37% of all neoplasms have transited through an intermediate tetraploid phase [26, 28–31]; and (4) the experimental demonstration that tetraploid (but not diploid) murine epithelial cells lacking the mouse homologue of tumor protein p53 (TP53, better known as p53) were able to generate chromosomically unstable tumors when injected in the flank of immunodeficient mice [18]. Of note, aneuploid cells generated from tetraploid cells often display elevated tumorigenicity [14, 16, 17, 19, 22].

Programmed changes in ploidy are believed to contribute to the development and homeostasis of a restricted panel of mammalian tissues or organs, including blood, liver, muscle, skin and placenta (reviewed in [12, 32]). In most but not all [33, 34] of these contexts, programmed polyploidy represents a terminally differentiated, non-cycling state [12, 32]. On the contrary, when occurring in proliferating cells, tetraploidy is normally sensed as a danger, leading to the activation of intrinsic, cell-autonomous processes such as cell cycle arrest [35–37], mitotic catastrophe [38], or regulated forms of cell death [39–41]. In addition, non-physiological tetraploidy can be detected and destroyed by the immunosurveillance system [42, 43]. The high incidence of aneuploid genomes in human neoplasms suggests that the barrier limiting the presence of tetraploid cells is bypassed during tumorigenesis. In line with this notion, the loss of tumor suppressor genes, including retinoblastoma 1 (RB1), p53 and adenomatous polyposis coli (APC), as well as the activation of oncogenes, including v-myc avian myelocytomatosis viral oncogene homolog (MYC), results in the generation of tetraploid cells (reviewed in [11]).

Tetraploidy could thus provide a handle by which to selectively eradicate the most aggressive cancer cells [44]. Most anti-tetraploid strategies designed so far are targeting intrinsic characteristics of tetraploid cells, such as their increased dependency upon accurate mitotic machinery, their elevated production of reactive oxygen species (ROS) and their extensive metabolic rewiring. Thus, tetraploids were proven to preferentially or selectively succumb to the inhibition of cell cycle or mitotic regulators, including checkpoint kinase 1 (CHEK1, best known as CHK1) [20], aurora kinase B (AURKB) [45], and kinesin family member 11 (KIF11, best known as EG5) [46], but also to antioxidants [47], and to perturbations of energy metabolism, as those provoked by overactivation of AMP-activated protein kinase (AMPK) [48], the glycolytic inhibitor 2-deoxyglucose [49], and mechanistic target of rapamycin (MTOR) inhibitors in combination with AURKB inhibitors [50, 51].

Increasing CIN by targeting mitotic regulators, including MPS1, a mitotic kinase involved in spindle assembly checkpoint (SAC) [52–55], has emerged as a valid approach to potently and preferentially kill cancer cells [56–63]. Here, we investigated the impact of MPS1 perturbation on the proliferation and survival of diploid *versus* tetraploid tumor cells, showing that the duplication of an entire set of chromosomes sensitizes cancer cells to MPS1 inhibition or depletion.

## RESULTS

### Effect of the abrogation of MPS1 function on tetraploid survival

**Figure 1:**
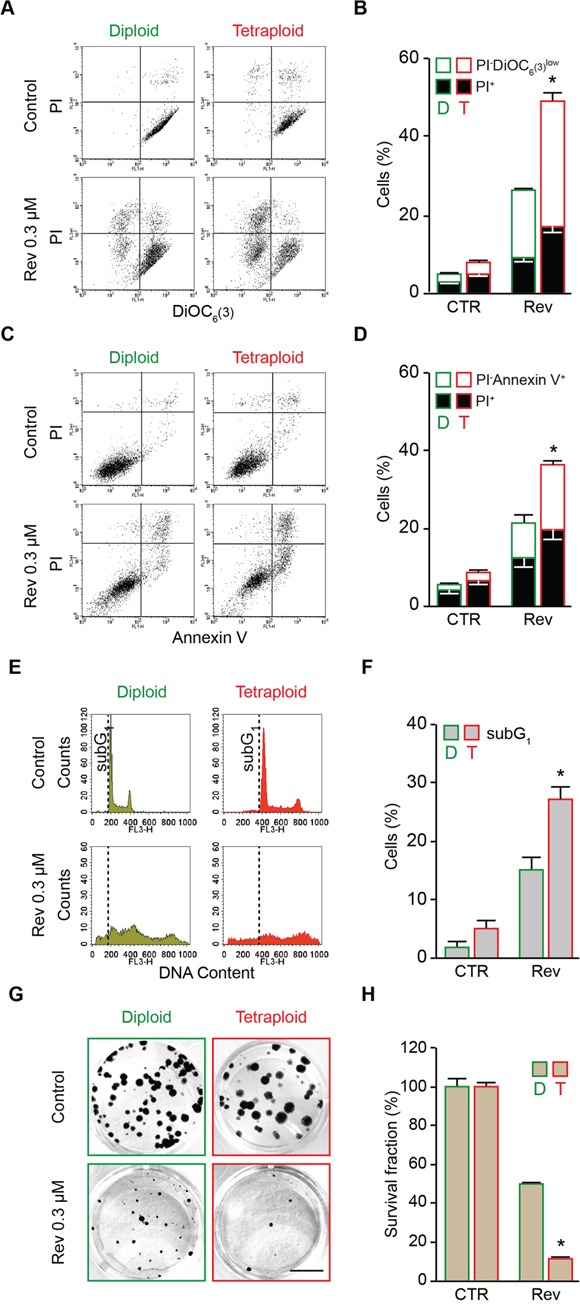
Preferential killing of tetraploid tumor cells by reversine-mediated MPS1 inhibition **A.** and **B.** Diploid and tetraploid human colorectal carcinoma HCT 116 cells (framed in green and red, respectively) were left untreated or treated for 72 hours (h) with 0.3 μM reversine and then co-stained with the vital dye propidium iodure (PI) and the mitochondrial membrane potential (Δψm)-sensing dye DiOC_6_(3) for the evaluation of cell death–associated parameters by cytofluorometry. Representative plots are showed in panel (A), while quantitative data are represented in panel (B). In panel (B) white and black columns depict the percentage of dying (PI^−^DiOC_6_(3)^low^) and dead (PI^+^) cells, respectively. **C.** and **D.** Diploid and tetraploid HCT 116 cells (framed in green and red, respectively) administered or not with 0.3 μM reversine for 72 h were stained for the cytofluorometric detection of phosphatidylserine exposure with Alexa Fluor 488-conjugated Annexin V. Representative dot plots and quantitative data are reported in panels (C) and (D), respectively. In panel (D) white columns depict the percentage of dying cells (PI^−^Annexin V^+^) while black columns illustrate dead cells (PI^+^). **E.** and **F.** Diploid and tetraploid HCT 116 cells (framed in green and red, respectively) left untreated or exposed for 72 h with 0.3 μM reversine were fixed with ethanol and labelled with the DNA dye PI, for the quantification of the hypodiploid, subG1 apoptotic population. Representative plots (E) and quantitative data (F) are reported. **G.** and **H.** Diploid and tetraploid HCT 116 cells (framed in green and red, respectively) seeded at low cell density were left untreated or exposed to 0.3 μM reversine for 24 h. Upon drug washout, cells were cultivated for 15 days in fresh, drug-free medium before crystal violet staining and colony counting. Representative images of the plates (scale bar = 1 cm) (G) as well as quantitative data obtained upon normalization to plating efficiency (H) are shown. In panel (B), (D), (F) and (H) data are reported as means ± SEM (n ≥ 3). *p < 0.001 (Mann–Whitney test), as compared with diploid subjected to the same treatment condition. CTR, control; diploid, D; tetraploid, T; reversine, Rev.

To evaluate the differential impact of MPS1 inhibition on the survival of cancer cells differing in their ploidy, we took advantage of a panel of diploid and tetraploid clones derived from parental human colon carcinoma HCT 116 and RKO cells, which we previously isolated and characterized [41], or from human malignant fibrous histiocytoma MFH152 cells, which we generated in this study by flow cytometry-assisted cloning [41]. These clones were left untreated or were administered with low doses (from 0.05 to 0.30 μM) of reversine, a small molecule that specifically inhibits MPS1 at submicromolar concentrations [64]. At the end of the treatment period, cell death was evaluated by flow cytometry-mediated measurement of well-recognized apoptotic parameters [65, 66], including dissipation of mitochondrial inner transmembrane potential (Δψm), phosphatidylserine (PS) surface exposure and DNA fragmentation (Figure 1 and Supplementary Figure S1). Δψm loss was measured on live cells (excluding the vital dyes propidium iodure, PI, or 4′,6-diamidino-2-phenylindole, DAPI) with either of the two Δψ_m_-sensitive dyes, dihexiloxalocarbocyanine iodide (DiOC_6_(3)) or tetramethylrhodamine methyl ester (TMRM). PS surface exposure was evaluated in live cells by staining with fluorophore-labeled Annexin V. DNA fragmentation was determined on fixed cells labeled with the DNA intercalating dye PI. As compared to their diploid counterparts, tetraploid HCT 116 (Figure 1A–1F and Supplementary Figure S1), RKO (Supplementary Figure S2A) and MFH152 (Supplementary Figure S2B) clones were particularly sensitive to reversine, as demonstrated by the elevated percentage of dying cells [displaying mitochondrial potential loss (PI^−^DiOC_6_(3)^low^ or DAPI^−^/TMRM^low^) or positivity for Annexin V (PI^−^Annexin V^+^)], dead cells [*i.e*., presenting plasma membrane permeabilization with a PI^+^ or DAPI^+^ phenotype], and cells with a hypodiploid (subG_1_) DNA content (Figure 1A–1F and Supplementary Figure S1). In line with these observations, submicromolar doses of reversine significantly reduced the clonogenic potential of tetraploid HCT 116 cells much more than that of diploid controls (surviving fraction of diploids *vs* tetraploids at 0.3 μM reversine: ∼12% *vs* ∼50%) (Figure 1G and 1H).

**Figure 2:**
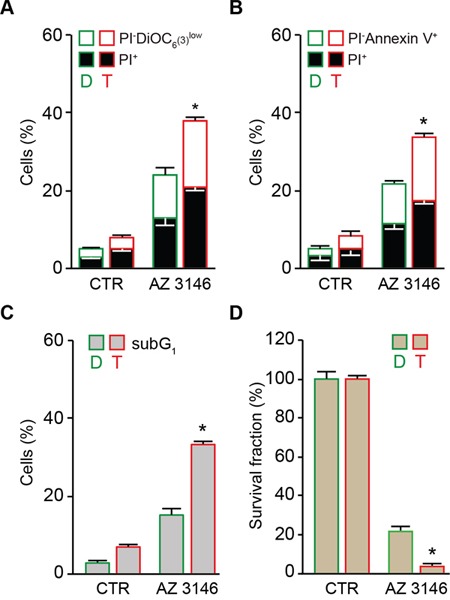
Preferential killing of tetraploid tumor cells by AZ 3146-mediated MPS1 inhibition **A.** and **B.** Diploid and tetraploid human colon carcinoma HCT 116 cells (framed in green and red, respectively) were left untreated or treated with 5 μM AZ 3146 for 72 hours (h) and then either co-stained with the vital dye propidium iodure (PI) and the mitochondrial membrane potential (Δψm)-sensing dye DiOC_6_(3) (A) or stained with the phosphatidylserine binding protein Annexin V conjugated to Alexa Fluor 488 for the evaluation of cell death–associated parameters by cytofluorometry. In panel (A) white and black columns depict the percentage of dying (PI^−^ DiOC_6_(3)^low^) and dead (PI^+^) cells, respectively. In panel (B) white and black columns illustrate the percentage of dying (PI^−^Annexin V^+^) and dead (PI^+^) cells, respectively. **C.** Diploid and tetraploid HCT 116 cells (framed in green and red, respectively) left untreated or exposed for 72 h with 5 μM AZ 3146 were fixed with ethanol and labelled with the DNA dye PI, for the quantification of the hypodiploid, subG1 apoptotic population. Quantitative data are reported. **D.** Diploid and tetraploid HCT 116 cells (framed in green and red, respectively) seeded at low cell density, were left untreated or exposed to 5 μM AZ 3146 for 24 h. Upon drug washout, cells were cultivated for 15 days in fresh, drug-free medium before crystal violet staining and colony counting. Quantitative data obtained upon normalization to plating efficiency are shown. In all the panels data are reported as means ± SEM (*n* ≥ 3). *p < 0.001 (Mann–Whitney test), as compared with diploid cells subjected to the same treatment condition. Diploid, D; tetraploid, T.

**Figure 3:**
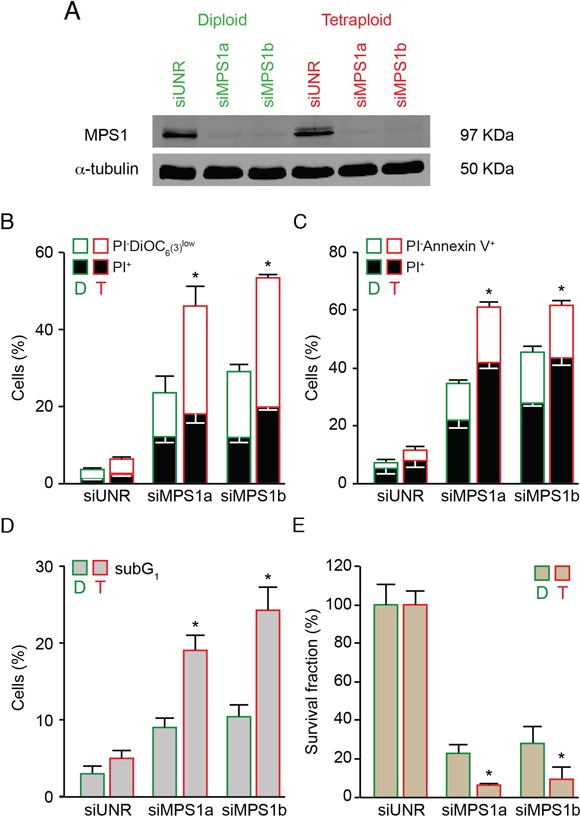
Increased sensitivity of tetraploid tumor cells to MPS1 depletion **A–C.** Diploid and tetraploid human colorectal carcinoma HCT 116 cells (framed in green and red, respectively) were transfected with an unrelated small interfering (si) RNA (siUNR) or two specific siRNAs directed against MPS1 (siMPS1a and siMPS1b). Upon 72 hours (h), cells were collected and lysed, then cell lysates were analyzed by western-blot using antibodies directed against MPS1 and α-tubulin (A). Alternatively, cells were subjected to the determination of the cell death–associated parameters by flow cytometry upon co-staining with the propidium iodure (PI) and DiOC_6_(3) dyes (B) or staining with Alexa Fluor 488-conjugated Annexin V (C). In panel (B) white and black columns illustrate the percentage of dying (PI^−^ DiOC_6_(3)^low^) and dead (PI^+^) cells, respectively. In panel (C) white and black columns illustrate the percentage of dying (PI^−^Annexin V^+^) and dead (PI^+^) cells, respectively. **D.** Diploid and tetraploid HCT 116 cells (framed in green and red, respectively) transfected for 72 h with siUNR, siMPS1a or siMPS1b were fixed with ethanol and labelled with PI for the quantification of the hypodiploid, subG1 apoptotic population. Quantitative data are reported. **E.** Clonogenic assay on diploid and tetraploid HCT 116 cells (framed in green and red, respectively) upon transfection with siUNR, siMPS1a and siMPS1b for 48 h followed by washout and cultivation in drug-free medium for further 15 days. Quantitative data obtained upon normalization to plating efficiency are shown. In panel (B), (C), (D) and (E) data are reported as means ± SEM (*n* ≥ 3). *p < 0.001 (Mann–Whitney test), as compared with diploid clones subjected to the same treatment condition. Diploid, D; tetraploid, T.

A similar preferential anti-tetraploid effect was observed in the HCT 116, RKO and MFH152 diploid/tetraploid pair with an alternative pharmacological inhibitor of MPS1 named AZ 3146 [67] (Figure 2 and Supplementary Figure S2) or in the HCT 116 diploid/tetraploid pair by depleting MPS1 via the transfection of small interfering (si) RNAs specifically directed against this kinase (siMPS1) (Figure 3), thereby ruling out potential off-target effects of reversine.

Altogether these findings demonstrate that abolishing MPS1 functions is an efficient strategy to preferentially and efficiently kill tetraploid cancer cells.

### Targeting MPS1 perturbs tetraploid cell cycle divisions

**Figure 4:**
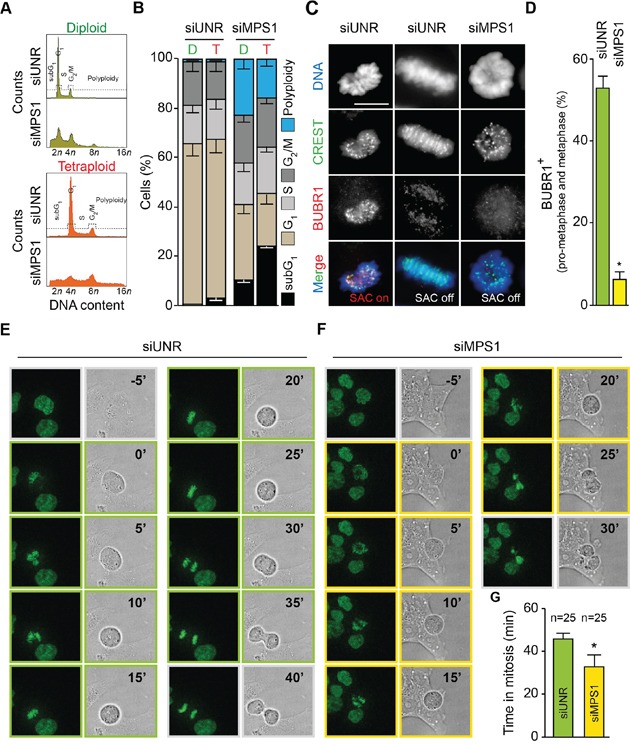
MPS1 abrogation shortens mitosis and abolishes SAC in tetraploid tumor cells **A.** and **B.** Diploid and tetraploid human colorectal carcinoma HCT 116 cells (framed in green and red, respectively) were transfected with an unrelated small interfering (si) RNA (siUNR) or a specific siRNA directed against MPS1 (siMPS1) for 72 hours (h) and then fixed and stained with propidium iodure for the cytofluorometric assessment of cell cycle progression. Cell cycle distribution analyzed by flow cytometry for diploid and tetraploid cells is displayed in panel (A). Quantitative data (means ± SEM; n = 3) of the corresponding flow cytometry profiles are plotted in panel (B). Diploid, D; tetraploid, T. **C.** and **D.** Tetraploid HCT 116 cells were transfected with siUNR or siMPS1 for 24 h, treated with 20 μM MG132 for 4 h (a relatively short time to avoid increasing the risk of putative side effects unrelated to mitosis) and then processed for the immunofluorescence-assisted detection of spindle assembly checkpoint (SAC) activation as indicated by the localization of BUBR1 at CREST-labelled kinetochores. In panel (C) representative microphotographs of a normal prometaphase (SAC on), a normal metaphase (SAC off), and an abnormal metaphase (SAC off) in cells transfected with the indicated siRNA are reported. Scale bar = 10 μm. In panel (D) representing quantitative data, columns illustrate the percentage of prometaphases plus metaphases exhibiting SAC activation. Data are reported as means ± SEM (*n* = 3). *p < 0.01 (two-tailed *t* test), as compared with siUNR transfected tetraploid cells. **E–G.** Tetraploid HCT 116 cells expressing a green fluorescent protein-tagged variant of histone 2B (H2B-GFP) chimera were transfected with siUNR or siMPS1 and then monitored by live videomicroscopy for approximately 24 h to determine the time spent in mitosis. Image were taken every 5 minutes. Representative snapshots of siUNR- and siMPS1-transfected tetraploid mitosis are shown in panel (E) and (F), respectively, while quantitative data (means ± SEM) are reported in panel (G) (*n* = 25). *p* < 0.01 (two-tailed *t* test), as compared with siUNR transfected tetraploid cells. Full-length movies are provided as Supplementary Movies 1–4.

When analyzing cell cycle profiles by flow-cytometry upon staining of fixed cells with the DNA dye PI, we observed that the inhibition or depletion of MPS1 provoked a dramatic perturbation of diploid and tetraploid cell cycle progression, including a moderate accumulation of cells with a DNA content of 4*n* (in diploid clones) and 8*n* (in tetraploid clones) and a major increase in the fraction of polyploid cells (DNA content > 4*n* and > 8*n* for diploid and tetraploid clones, respectively) (Figures 1E, 4A and 4B; Supplementary Figure S1C and 1E). Of note, the extent of polyploidization induced by MPS1 depletion was higher in diploid than tetraploid clones (Figure 4B and Supplementary Figure S1B–S1E). We then evaluated the impact of MPS1 abrogation on the regulation and timing of tetraploid mitosis. As illustrated in Figure 4C and 4D, upon MPS1 depletion the percentage of prometaphases plus metaphases displaying the kinetochore localization of the SAC component BUB1 mitotic checkpoint serine/threonine kinase B (BUB1B, better known as BUBR1) dropped drastically close to zero, thus indicating the complete abrogation of SAC function. In line with this evidence, the pharmacological inhibition or RNA interference-mediated depletion of MPS1 prevented the mitotic block imposed by antimitotic agents as demonstrated by the reduction of the fraction of phosphorylated histone H3 (pH3) positive cells (Supplementary Figure S3). To corroborate these findings, we performed videomicroscopy analyses on tetraploid HCT 116 cells engineered to stably express a green fluorescent protein-tagged variant of histone 2B (GFP-H2B), which allows for the intravital visualization of chromatin and chromosomes. The depletion of MPS1 significantly reduced the time spent by tetraploid cells in mitosis (average of 45.8 ± 2.8 minutes when transfected with an unrelated siRNA *vs* 32.8 ± 5.4 minutes when siMPS1-transfected) (Figure 4E–4G, and Supplementary Movie S1–S4). These results are consistent with the reduction of mitosis duration upon MPS1 inhibition in human osteosarcoma U2OS cell lines [68] and demonstrate that MPS1 contributes to the correct timing and execution of mitosis regardless of the cell ploidy status.

**Figure 5:**
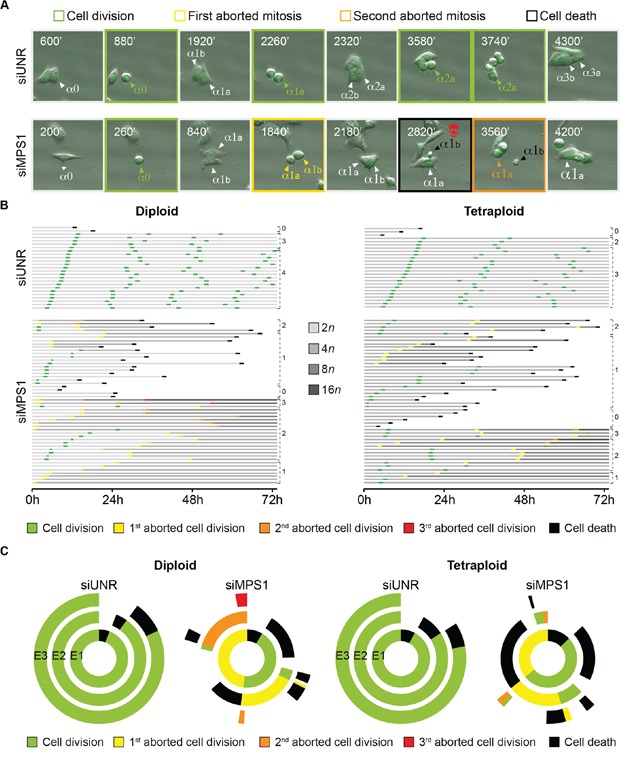
Cell fate profiling of diploid and tetraploid tumor cells depleted of MPS1 **A–C.** Diploid and tetraploid human colorectal carcinoma HCT 116 cells stably expressing a green fluorescent protein-tagged variant of histone 2B (H2B-GFP) chimera were transfected with an unrelated small interfering (si) RNA (siUNR) or a specific siRNA directed against MPS1 (siMPS1) and then monitored by live videomicroscopy for 72 hours (h). Representative snapshots of tetraploid cells transfected as indicated are shown in panel (A), whereas single cell fate profiles of siUNR- or siMPS1-transfected diploid (*n* = 25) and tetraploid (*n* = 50), and transgenerational cell fate profiles of siUNR or siMPS1 transfected cells (*n* >100) are depicted in panel (B) and (C), respectively. In panel (A) alphas (α) indicate individual cells, whose sequential cell divisions are numbered with “0”, “1”,“2” and “3”. The two daughter cells of a bipolar division are depicted with “a” and “b”; while the single cell derived by an abortive division is indicated by an increase in the size type. The snapshots showing successful cell divisions are framed in green, while those showing the first and second abortive cell divisions are framed in yellow and orange, respectively. The snapshot framed in black highlights the death of one cell following an abortive mitosis. In panel (B) horizontal columns represent single cells over the time, as indicated in hours. The color code depicting successful (green) or aborted (yellow, orange and red) cell divisions, as well as cell death (black) is used as in panel (A). The increase in cell ploidy following abortive cell division is represented by grey darkening, while numbers indicate mitotic events (*i.e*., successful or abortive cell division). Please note that cell divisions were considered to be successful only when daughter cells were clearly separated. In panel (C) concentric circles depict three consecutive generational events (E), starting from E1 (inner circle), using the same color code as used in panel (A) and (B). Full-length movies are provided as Supplementary Movies 5 and 6.

To further characterize the mechanism of tetraploid tumor cell killing by MPS1 abrogation, we depleted MPS1 in GFP-H2B diploid and tetraploid clones and followed them by videomicroscopy for 72 hours (h). This analysis confirmed that the knockdown of MPS1 impairs both diploid and tetraploid mitoses (Figure 5A and 5B; Supplementary Movie S5 and S6). In particular, the depletion of MPS1 resulted in aborted cell divisions (1^st^ event depicted in yellow in the single cell fate profiles) generating a single daughter cell with a duplicated genome (Figure 5B). Alternatively, MPS1-depleted cells underwent a bipolar cell division (1^st^ event depicted in green in the single cell fate profiles) generating 2 daughter cells, which either entered an aborted mitosis (2^nd^ event depicted in yellow in the single cell fate profiles) or died during the following interphase (2^nd^ event depicted in dark in the single cell fate profiles) (Figure 5B). By comparing their transgenerational cell fate profiling, a representation in which the destiny of a cellular population is monitored across consecutive generations and events (E1, E2 and E3) are symbolized by a centripetal sequence of concentric ring segments [69], we confirmed that the incidence of abortive mitoses (*i.e*., the induction of polyploid cells) upon MPS1 depletion was lower in tetraploid than in diploid clones (Figure 5C, see also Figure 4B). Moreover, in the majority of the cases (75%), polyploids generated from diploid clones (depicted in yellow in the inner circle; siMPS1 condition) remained inert or underwent one or even two additional round(s) of aberrant/abortive mitosis (depicted in orange and red in the central and external circle, respectively; siMPS1 condition) with only 25% undergoing cell death (depicted in dark in the central circle; siMPS1 condition) (Figure 5C). On the contrary, a large fraction of polyploids generated from tetraploid clones (depicted in yellow in the inner circle; siMPS1 condition) died during the interphase that followed the first aborted mitosis (depicted in dark in the central circle; siMPS1 condition) (Figure 5C). These findings confirm the antitetraploid effect of MPS1 inhibition underscoring the relative incapability of tetraploid (as compared to diploid) cells to tolerate any further increase in ploidy in the absence of SAC.

Altogether these results indicate that targeting MPS1 preferentially kills tetraploid tumor cells by abolishing SAC function, eventually triggering an uncontrolled and lethal polyploidization program.

### Molecular mechanisms underlying the antitetraploid effect of MPS1 abrogation

**Figure 6:**
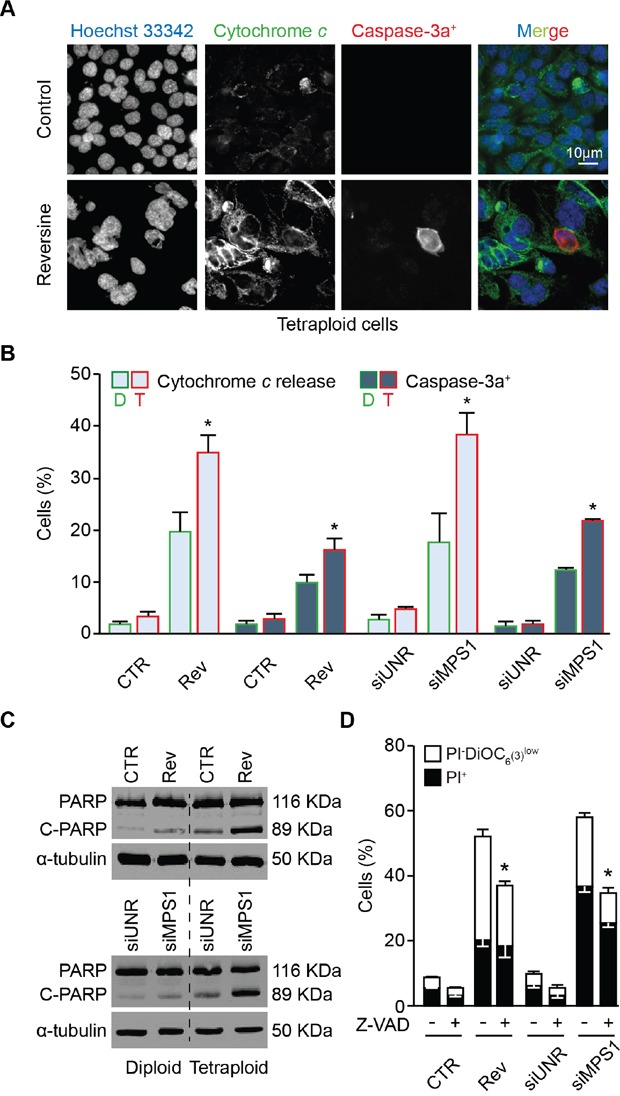
Mechanisms of cell death induced by MPS1 abrogation **A.** and **B.** Diploid and tetraploid human colorectal carcinoma HCT 116 cells (framed in green and red, respectively) treated or not with 0.3 μM reversine (A and B) or transfected with an unrelated small interfering (si)RNA (siUNR) or a specific siRNA directed against MPS1 (siMPS1) for 72 hours (h) (B) were stained to visualize cytochrome *c* (green fluorescence), activated caspase-3 (caspase-3a^+^, red fluorescence) and nuclei (Hoechst 33342, blue fluorescence) and analyzed by fluorescence microscopy. The percentage of cells exhibiting diffuse (as opposed to punctuate) cytochrome *c* staining or caspase-3 activation was determined as described in Materials and Methods. Representative fluorescence microphotographs of tetraploid cells (A) and quantitative results (means ± SEM, n = 3) for both diploid and tetraploid clones (B) are shown. *p < 0.001 (Mann–Whitney test), as compared with diploid cells subjected to the same treatment or transfection condition. **C.** Western-blot analysis of protein extracts from diploid and tetraploid HCT 116 cells treated or not with 0.3 μM reversine or transfected with siUNR or siMPS1 for 72 h using an antibody directed against poly (ADP-ribose) polymerase (PARP) and recognizing the cleaved (C-PARP, 89 KDa) and uncleaved (PARP, 116 KDa) forms. Alpha-tubulin was used to verify equal loading. **D.** Tetraploid HCT 116 cells were treated or not for 72 h with 0.3 μM reversine or transfected with the indicated siRNAs alone or in combination with 25 μM Z-Val-Ala-Asp-fluoromethylketone (Z-VAD-fmk, abbreviated as Z-VAD), followed by co-staining with DiOC_6_(3)/propidium iodure (PI) and cytofluorometric analysis. White and black columns illustrate the percentage of dying (PI^−^DiOC_6_(3)^low^) and dead (PI^+^) cells, respectively. Data are reported as means ± SEM (*n* = 3). *p < 0.001 (Mann–Whitney test), as compared with cells subjected to the same treatment or transfection condition but not exposed to Z-VAD. CTR, control; diploid, D; tetraploid, T; reversine, Rev.

We thus investigated the mechanisms involved in the execution of mitotic catastrophe induced by MPS1 inhibition or depletion in tetraploid cells. As shown by videomicroscopic analyses, the death of tetraploid tumor cells succumbing upon MSP1 inhibition was always preceded by chromatin condensation (pyknosis) and nuclear fragmentation (karyorrhexis), two morphological hallmarks of apoptosis [66] (Figures 5A and 6A, and Supplementary Movie S6). Moreover, tetraploid cell death triggered by MPS1 inhibition or depletion displayed the classical features of the intrinsic pathway of apoptosis, including the release of the pro-apoptotic factor cytochrome *c* from mitochondria to the cytosol and the activation of caspase-3 (Figure 6A and 6B). Of note, the percentage of both cytochrome *c* release and caspase-3 activation was higher in tetraploid clones than in their diploid counterparts, a result that is in line with the preferential sensitivity of tetraploid cells to MPS1 inhibition/depletion. Further confirming the role of caspases in the execution of apoptosis, tetraploid cells (but less so their diploid counterparts) responding to reversine or siMPS1 manifested the apoptosis-associated cleavage of poly (ADP-ribose) polymerase (PARP) (Figure 6C). Moreover, the pre-administration of the broad-spectrum caspase inhibitor Z-Val-Ala-Asp-fluoromethylketone (Z-VAD-fmk) significantly reduced the death of tetraploid cells responding to the inhibition or depletion of MPS1 (Figure 6D). Recently, we and others reported some cooperations between polo-like kinase 1 (PLK1) and MPS1 in SAC initiation [70, 71]. In line with this evidence, not only the depletion of MPS1 but also that of PLK1 had a preferential cytotoxic effect on tetraploid as compared to diploid cancer cells (Supplementary Figure S4). Of note, the co-depletion of MPS1 and PLK1 did not further increase the level of cell death, while the knock-down of MPS1 sensitized tumor cells to sublethal doses of paclitaxel independently of the basal ploidy level (Supplementary Figure S4). Altogether, these results indicate that, in tetraploid cells, MPS1 inhibition triggers mitotic catastrophe that is executed by the intrinsic pathway of apoptosis.

## DISCUSSION

In this study we developed a novel strategy for targeting tetraploid tumor cells based on the abrogation of the mitotic kinase MPS1. By employing different pairs of tumor clones generated from the same parental cell lines and displaying distinct levels of ploidy (*i.e*., diploid *vs*. tetraploid) we provided strong evidence that the depletion or inhibition of MPS1 potently kills tetraploid cancer cells (and to a lesser extent diploid cancer cells) via a mechanism involving the induction of mitotic catastrophe following aberrant or aborted cell divisions, and the activation of a mitochondrion- and caspase-dependent pathway of regulated cell death.

It is becoming increasingly clear that measures inducing an exaggerated level of CIN or an unscheduled hyper-polyploidization program may constitute valid antineoplastic strategies, potentially sparing non-tumor cells and/or selectively targeting cancers according to their genetic background [58–61, 63, 72–74]. The approaches developed so far are based on the rationale that tumor cells (1) frequently display defects in cell cycle checkpoints and thus could progress in their cell cycle even in the presence of gross alterations as those provoked by mitosis-perturbing and/or CIN-inducing agents, and (2) may survive only until a certain threshold level of CIN and/or ploidy. Here we demonstrated that MPS1 inhibition kills both diploid and tetraploid cancer cells, though with a preferential action on tumor cells that have undergone whole-genome duplication. The tumorigenic potential of tetraploid cells arises from their intrinsic high level of genomic instability [12, 22], and/or their capability to tolerate and survive the burden of continuous karyotype changes [26]. In this context, we surmise that the peculiar sensitivity of tetraploid tumors to MPS1 inhibition is not necessarily linked to their basal level of CIN but rather arises from (1) the elevated dependency of tetraploids on the activity of the SAC, which is functional and overactivated in these cells and whose abrogation affects their survival more potently than the survival of their diploid counterpart [20], and (2) the incapability of tetraploids to tolerate a further increase in ploidy in absence of SAC functions, a condition that *de facto* exaggerates CIN to a level incompatible with life. These findings are in line with the lack of correlation between the pattern of genomic instability (*i.e*., microsatellite instability *vs.* CIN) and the sensitivity to MPS1 inhibitors, and with the involvement of an obligatory step of tetraploidization in cancer killing induced by MPS1 inhibitors [59]. Emerging evidence ascribes non-mitotic functions to SAC components [75, 76]. Given our results, it is tempting to speculate that SAC activity may play a role in the increased tolerance to chromosome aberrations of tetraploids, which has been recently associated to tumor evolution [26]. Videomicroscopic analyses demonstrated that, in the absence of SAC, newly-generated octaploid cells died during the interphase that follows the aborted mitosis of tetraploid cells. Further studies are required to elucidate whether higher-order ploidy (*i.e*., more-than-tetraploidy) intrinsically triggers a specific apoptotic program and, if so, whether the absence of SAC function may be the signal responsible for the activation of this cascade.

MPS1 is considered among the most promising targets for cancer therapy for multiple reasons, including: (1) the low frequency of mutations of *MPS1* detected in tumors (reviewed by [52]); (2) the upregulation of *MPS1* found in a variety of human cancers [55, 57, 77–83] often correlated to CIN/aneuploidy [4, 9, 84–86] or high tumor grade and aggressiveness [58, 87, 88]; (3) the pleiotropic roles of MPS1 in distinct phases of the cell cycle [52, 62, 89–100]; (4) the potential selectivity of MPS1 inhibitors toward malignant *versus* normal or immortalized cells [56–58, 60–62]; and (5) the synergy between MPS1 inhibitors and conventional anticancer agents, including antimitotics [59, 61, 87] and radiotherapy [89].

Here, we provide an extra point in support of the therapeutic utility of MPS1 inhibitors, showing that MPS1 depletion or inhibition kills tetraploid cancer cells more efficiently than diploid cancer cells. This may be relevant in the context of cancer therapy given that tetraploid cells are believed to promote tumorigenesis and also display high resistance to conventional anticancer regimens such as DNA damaging agents.

Some clinical trials have been launched to assess the safety and therapeutic profile of specific MPS1 inhibitors in cancer patients (source: https://clinicaltrials.gov/). It appears thus of interest to confirm the potential antitetraploid effect of these compounds either in mouse models of spontaneous tetraploidization-driven tumorigenesis [48] or in clinical samples, by evaluating the ploidy status of tumors responding to MPS1 inhibition.

## MATERIALS AND METHODS

### Cell lines and culture conditions

Diploid and tetraploid clones derived from human colon carcinoma HCT 116 cells were routinely maintained in McCoy's 5A medium supplemented with 10% fetal calf serum (FCS), 10 mM HEPES buffer, 100 units/mL penicillin G sodium and 100 μg/mL streptomycin sulfate (all provided from Thermo Fisher Scientific-Gibco, Waltham, MA). Human malignant fibrous histiocytomas MFH152 cells as well as diploid and tetraploid clones derived from these cell lines were cultured in Dulbecco's modified Eagle's medium (DMEM) supplemented with 10% FCS and 100 units/mL penicillin G sodium (Thermo Fisher Scientific-Gibco). Diploid and tetraploid HCT 116 and RKO clones transfected with a cDNA coding for a histone 2B-green fluorescent protein (H2B-GFP) (from BD Biosciences-PharMingen, San Jose, CA) were grown in McCoy's 5A medium supplemented as above plus 20 μg/mL blasticidine (Thermo Fisher Scientific-Gibco). Cells were seeded onto the appropriate supports (6-, 12-, 24- or 96-well plates) 24 h before the beginning of experiments.

### Chemicals

MG132, paclitaxel, nocodazole, and reversine were purchased from Sigma-Aldrich (St. Louis, MO) and stocked as a 10 mM solution in dimethyl sulfoxide (DMSO). AZ 3146 was obtained from Tocris (Bristol, United Kingdom) and stocked as a 10 mM solution in DMSO. The pan-caspase inhibitor z-VAD-fmk was obtained from Bachem Bioscience (Bubendorf, Switzerland) and stocked as a 50 mM solution in N,N-dimethylformamide (DMF). The appropriate amount of DMSO and/or DMF was always employed for negative control conditions.

### RNA interference

Diploid and tetraploid HCT 116 cells were seeded at low density in 6-, 12- or 96-well plates and after 24 h transfected with an unrelated siRNA (siUNR), two specific siRNAs directed against MPS1 mRNAs (siMPS1a and siMPS1b) (all purchased from Eurogentec, Liege, Belgium) or a specific siRNA directed against PLK1 mRNAs (siPLK1, ON-TARGETplus SMARTpool, L-003290–00-0005; GE Dharmacon, Lafayette, CO) by means of oligofectamine RNAiMAX transfection reagent (Thermo Fisher Scientific-Invitrogen), according to the manufacturer's instructions. The following siRNAs were used: 5′-GCCGGUAUGCCGGUUAAGUdTdT-3′(siUNR), 5′-UGGUUGAGUUUGUUGCUCAUUdTdT-3′(siMPS1a), and 5′-CCCAGAGGACUGGUUGAGUd TdT-3′(siMPS1b).

### Cytofluorometric studies

For the quantification of apoptotic features, the assessment of cell cycle distribution, and the simultaneous measurement of DNA content and phosphorylated histone H3 (pH3) levels were performed as reported [48, 73]. The following antibodies were used: primary anti-phosphorylated histone H3 antibody (rabbit polyclonal IgG1 #06–570; Millipore-Chemicon International, Temecula, CA) and Alexa Fluor 488 goat anti-rabbit secondary antibody (Thermo Fisher Scientific). Phosphatidylserine exposure was quantified using the Alexa Fluor 488 Annexin V/Dead Cell Apoptosis Kit (Thermo Fisher Scientific-Invitrogen). Cytofluorometric acquisitions were performed by means of a FACSCalibur (BD Biosciences) or a FACSCanto (BD Biosciences) cytofluorometer, while data analysis was conducted using the CellQuestTM software (BD Biosciences). Only the events characterized by normal forward scatter (FSC) and side scatter (SSC) parameters were gated for inclusion in the statistical analysis.

### Clonogenic survival assay

The clonogenic assay was performed and analyzed as reported above [73]. Briefly, cells were seeded at low concentrations and were left untreated, treated with MPS1 inhibitors or transfected with siUNR or siMPS1 for further 24 h followed by washout and culture in standard conditions for up to 15 days. Colonies were then fixed/stained with aqueous crystal violet and counted.

### Immunoblotting

For the detection of protein levels, cells were harvested, washed with PBS and lysed for 30 min on ice in a buffer prepared in 50 mM Tris (pH 7.4) and containing 250 mM NaCl, 0.1% NP-40, 0.1 mM phenylmethylsulfonyl fluoride (PMSF), aprotinine at 10 mg/mL, leupeptine at 10 mg/mL, and 100 mM NaF. Cell lysates were then centrifuged for 10 min at 13 000 rpm and the concentration of soluble proteins in supernatant was measured by the Bradford method. Equal amount of proteins (30 μg) were resolved by SDS/PAGE and electro-transferred onto nitrocellulose membrane, which was then incubated overnight with the appropriate primary antibody. Thereafter, membranes were incubated for 1 h at room temperature with the appropriate DyLight 800 conjugate secondary antibody (Thermo Fisher Scientific-Pierce antibodies) and revealed with the LI-COR Odyssey^®^ scanner and software (LI-COR Biosciences, Lincoln, NE). The following antibodies were used: α-tubulin (mouse monoclonal IgG1, #T9026; Sigma-Aldrich), MPS1 (mouse monoclonal IgG1 #ab11108; Abcam, Cambridge, UK) and PARP (mouse monoclonal IgG1 #9542; Cell Signaling Technology, Billerica, MA).

### Immunofluorescence microscopy

For detection of apoptotic markers and SAC activation, cells were fixed with 4% PFA (in PBS), permeabilized with 0.1% Triton X-100 (in PBS), and immunostained with antibodies directed against cytochrome *c* (1/100 dilution; #6H2.B4, BD Biosciences), cleaved caspase-3 (Asp175) (1/200 dilution; #9661, Merck Millipore-Cell Signaling Technology), BUBR1 (1/200 dilution; mouse monoclonal IgG1 #612502, BD Biosciences) or human anti-nuclear-centromere CREST (Europa Bioproducts, Cambridge, UK). Revelation was performed with the appropriate Alexa Fluor conjugated secondary antibodies (Thermo Fisher Scientific-Invitrogen). Hoechst 33342 (1 μg/mL; BD Biosciences) was used for nuclear counterstaining. Images were captured with a Zeiss AxioimagerZ1 motorized microscope (Zeiss, Oberkochen, Germany) driven by Axiovision software (Zeiss) and analyzed with the open source software Image J (freely available from the National Institute of Health, http://rsb.info.nih.gov/ij/).

### Videomicroscopy

For videomicroscopy, diploid and tetraploid H2B-GFP HCT 116 cells were grown in appropriate plates. The recording of the images started at the beginning of the treatment (t = 0) and images were taken every 5 min (Supplementary Movies S1–S4) for 24 h using a confocal spinning disk CSU-X1 Andor Nikonor (Andor Technology, Belfast, UK) coupled with Ti Eclipse microscope (Nikon, Tokyo, Japan) and driven by iQ3 software (Andor) or every 20 min (Supplementary Movies S5 and S6) for 72 h with a Leica DMIRE2 automated live cell microscope with a LMC 20 × 0.4 lens and appropriate filters (Leica, Wetzlar, Germany). Both transmitted light and fluorescence imaging were used to detect the cells. Images were analyzed with the open-source software Image J.

### Statistical procedures

Unless otherwise specified, all experiments were performed and independently repeated at least three times. In order to evaluate if the differences observed between diploid and tetraploid cell lines subjected to a given tratment were significantly larger than those observed in control (*i.e*., untreated) conditions, results from diploid and tetraploid clones were randomly paired to compute differences. Thereafter, these differences were compared between treatment and control conditions using one-tail Wilcoxon-Mann-Whitney test. One hundred random pairings were performed and the 100 obtained *p*-values were corrected for multiple testing using Benjamini-Hochberg method. Then, the number of corrected *p*-values lower than 5% (N) was computed. The higher this number the more probable the difference between treatment and control. The significance of N was then assessed using a resampling method allowing to estimate its distribution under the null hypothesis (that is “differences between diploid and tetraploid were equivalent between treatment and control”) and to compare the N value to this distribution. Hence, the computation of an empirical *p*-value allowed to identify situations where differences observed between diploid and tetraploid cell lines are significantly larger when applying a given treatment than in conditions. A significance threshold of 5% has been chosen but all significant tests exhibited *p*-values lower than 0.1%. Data were analyzed using “R” software (R Foundation for Statistical Computing, Vienna, Austria; HYPERLINK “http://www.R-project” http://www.R-project.org/). Two-tailed *t*-tests were performed with GraphPad Prism (GraphPad Software, Inc. La Jolla, CA) when conditions (treated/untreated) could be compared within a single cell line (Figure 3). Calculations exhibiting *p*-value < 0.01 were considered as statistically significant and indicated by an asterisk (*). Means ± SEM are represented.

## SUPPLEMENTARY MATERIALS METHODS FIGURES AND MOVIES



## Abbreviations

CIN: chromosome instability
FCS: fetal calf serum
GFP: green fluorescent protein
H2B: histone 2B
MPS1: monopolar spindle 1
PARP: poly (ADP-ribose) polymerase 1
pH3: phosphorylated H3
PLK1: polo-like kinase 1
PS: phosphatidylserine
SAC: spindle assembly checkpoint
siRNA: small interfering RNA
UNR: unrelated
Z-VAD-fmk: Z-Val-Ala-Asp-fluoromethylketone.
