# Supplementary material for: Whole-genome duplication increases tumor cell sensitivity to MPS1 inhibition
Source: Oncotarget. 2015 Nov 30;7(1):885–901. doi: 10.18632/oncotarget.6432 (PMC4808040; doi:10.18632/oncotarget.6432)
Supplement: Supplementary file 1 [file oncotarget-07-0885-s001.pdf]

SUPPLEMENTARY MATERIALS AND METHODS

Generation of diploid and tetraploid MFH152 clones

Diploid and tetraploid cells were generated as reported above [1]. Briefly, individual cells from the parental MFH152 cell line were cloned in 96-well plates (for a total of 10 plates) by using a FACSAria cell sorter from BD Biosciences. After 4 weeks of cultivation in standard conditions 50 clones were obtained and amplified. Of these clones, 5 were stable tetraploid and 30 stable diploid, as evaluated by flow-cytometry analyses upon staining with propidium iodure (PI) (Sigma-Aldrich, St. Louis, MO). Only early passage (<10) clones were employed in the study.

REFERENCES

1. Castedo M, Coquelle A, Vivet S, Vitale I, Kauffmann A, Dessen P, Pequignot MO, Casares N, Valent A, Mouhamad S, Schmitt E, Modjtahedi N, Vainchenker W, Zitvogel L, Lazar V, Garrido C, et al. Apoptosis regulation in tetraploid cancer cells. EMBO J. 2006; 25:2584–2595.

SUPPLEMENTARY FIGURES AND MOVIES

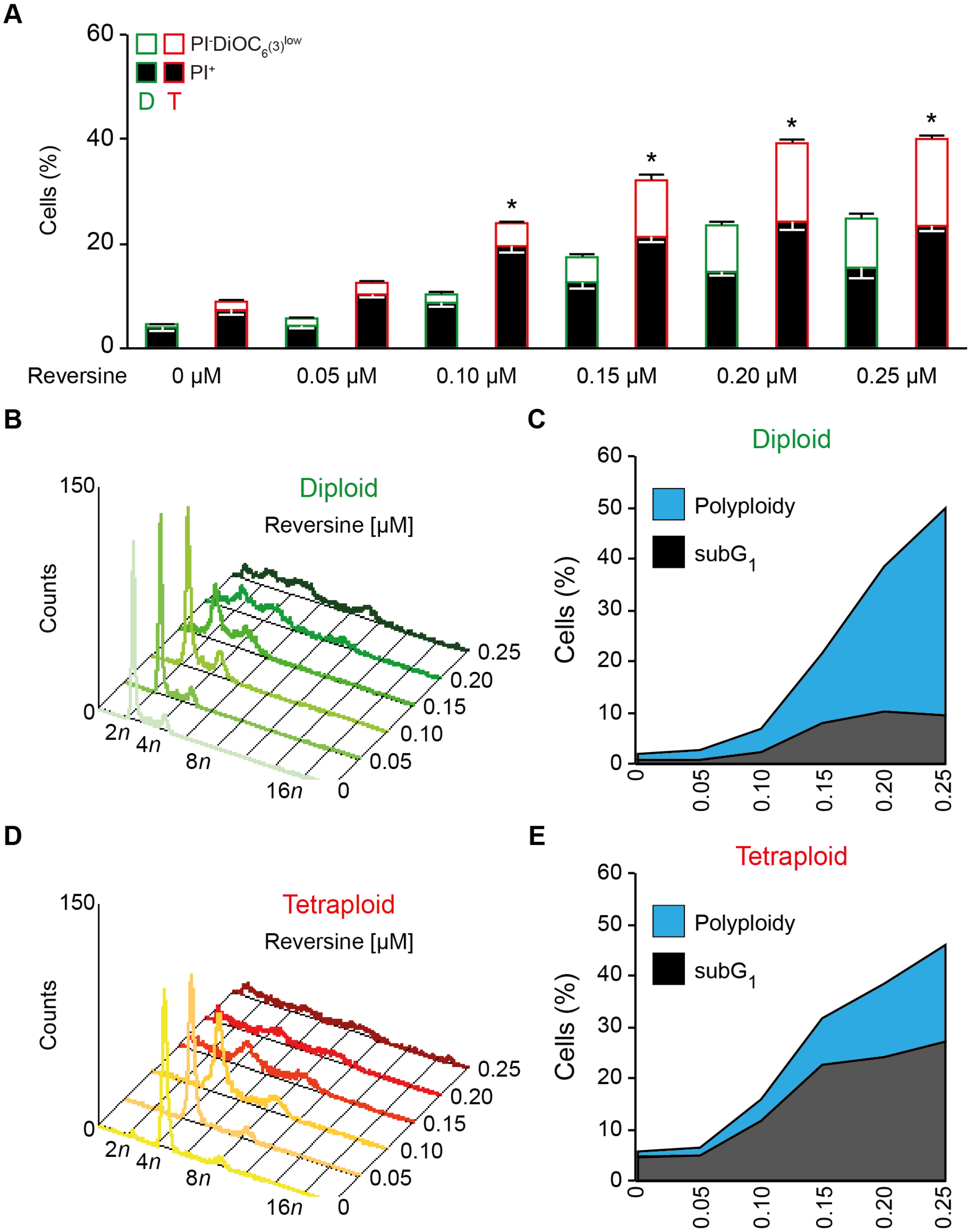

Supplementary Figure S1: Preferential killing of tetraploid tumor cells by low doses of reversine. A–E. Diploid and tetraploid human colon carcinoma HCT 116 cells (framed in green and red, respectively) were left untreated or treated with the indicated concentration of reversine for 72 hours (h) and then co-stained with the vital dye propidium iodure (PI) and the mitochondrial membrane potential (Δψm)-sensing dye DiOC$_6$(3) for the evaluation of cell death–associated parameters by cytofluorometry (A), or fixed with ethanol and labelled with PI, for the cytofluorometric assessment of cell cycle progression (B-E). In panel (A), white and black columns illustrate the percentage of dying (PI$^-$DiOC$_6$(3)^{low}) and dead (PI$^+$) cells (mean ± SEM; n = 3). Diploid, D; tetraploid, T. In panels (B) and (D), cell cycle flow cytometry profiles are showed while the quantification of the hypodiploid (subG$_1$ and apoptotic) population and the polyploid population is displayed in panels (C) and (E).

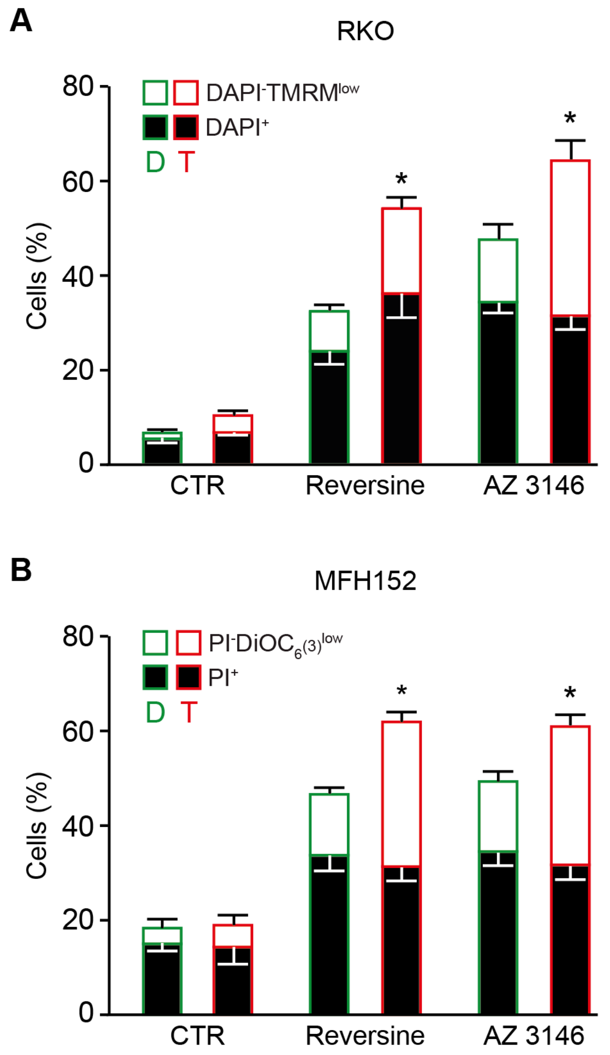

Supplementary Figure S2: Increased sensitivity of tetraploid tumor cells to MPS1 inhibitors. A, B. Diploid (framed in green) and tetraploid (framed in red) human colon carcinoma RKO cells (A) or diploid and tetraploid human malignant fibrous histiocytoma MFH152 clones (B) left untreated or administered with 0.3 µM reversine or 5 µM AZ 3146 for 72 hours (h) (RKO) or 120 h (MFH152) were co-stained with the vital dye 4′,6-diamidino-2-phenylindole (DAPI) and the mitochondrial transmembrane potential (Δψm) tetramethylrhodamine methyl ester (TMRM) (A) or with the vital dye propidium iodure (PI) or the Δψ-sensing dye dihexiloxalocarbocyanine iodide (DiOC$_6$6(3)) (B) for the evaluation of cell death–associated parameters by cytofluorometry. The percentage of dying cells (DAPI$^-$/TMRM^{low}) or (PI$^-$ DiOC$_6$(3)^{low}), and dead cells (DAPI$^+$) or (PI$^+$) are represented by white and black columns, respectively. Data are reported as means ± SEM ($n \geq 3$). *$p < 0.001$ (Mann–Whitney test), as compared with diploid subjected to the same treatment condition. Diploid, D; tetraploid, T.

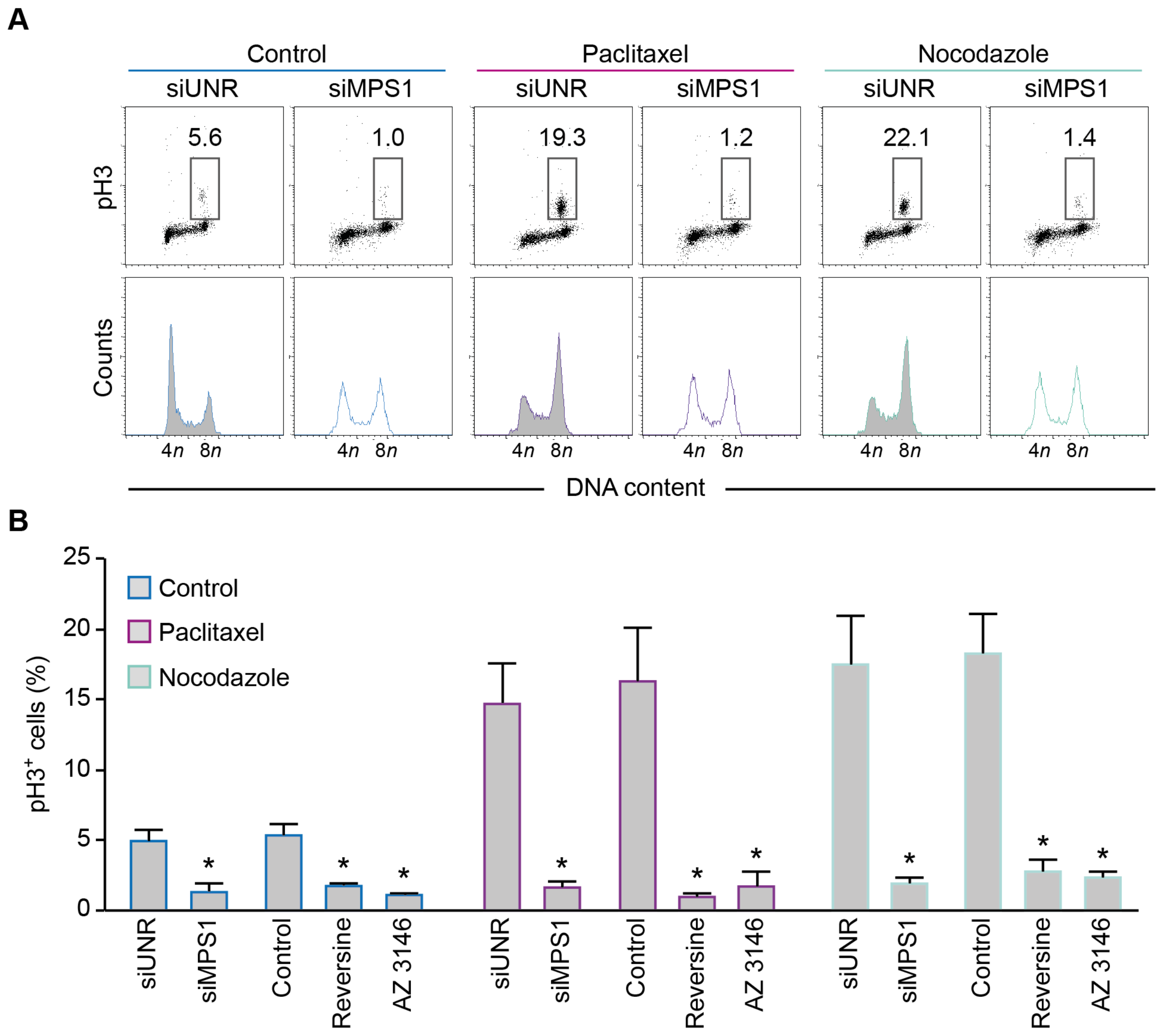

Supplementary Figure S3: Role of MPS1 in SAC function of tetraploid tumor cells. A, B. Tetraploid human colon carcinoma HCT 116 cells were left untreated, treated with 0.3 μM reversine, exposed to 5 μM AZ 3146, transfected with an unrelated small interfering (si) RNA (siUNR) or transfected with specific siRNA directed against MPS1 (siMPS1). After 4 hours (h) cells were administered or not with 100 ng/mL of nocodazole or paclitaxel for 15 h and then fixed with ethanol, immunostained with a specific antibody recognizing the mitotic marker phosphorylated histone H3 (pH3) and stained with propidium iodure (PI) for the simultaneous cytofluorometric assessment of cell cycle distribution and of the levels of pH3. Representative cell cycle distributions and scatter plots are shown in panel (A), while quantitative results are reported in panel (B). In panel (A) numbers indicate the percentage of cells found in each gate. Data are reported as means ± SEM ($n = 3$). *$p < 0.001$ (Mann–Whitney test), as compared with untreated cells (for reversine and AZ 3146) or siUNR-transfected cells (for siMPS1).

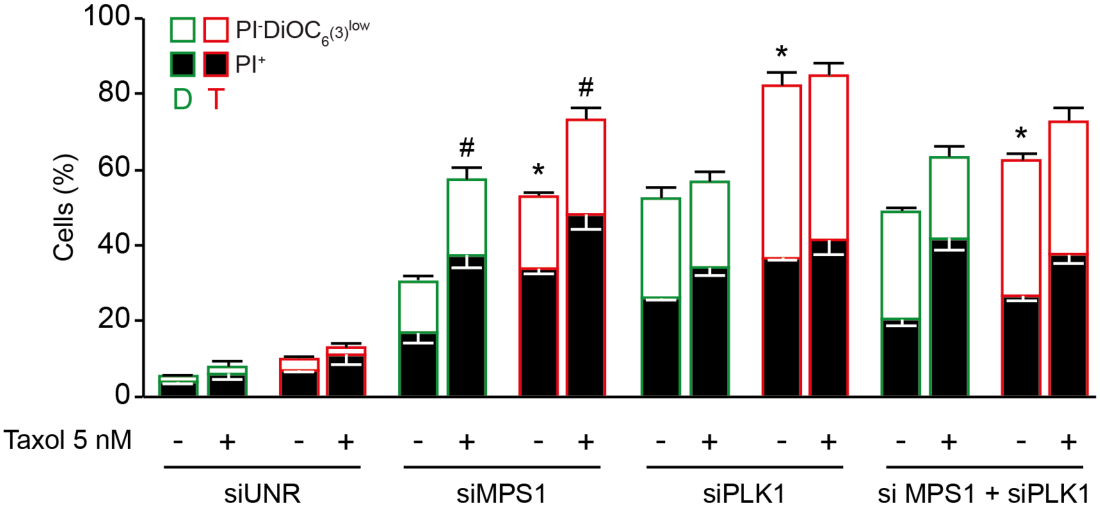

Supplementary Figure S4: Impact of PLK1 depletion on tetraploid cancer cell survival. Diploid and tetraploid human colon carcinoma HCT 116 cells (framed in green and red, respectively) were left untreated or treated with 5 nM of paclitaxel (PXL) and then transfected with an unrelated small interfering (si) RNA (siUNR) or a specific siRNA directed against MPS1 (siMPS1) or PLK1 (siPLK1). After 72 hours (h), cells were collected and co-stained with the vital dye propidium iodure (PI) and the mitochondrial membrane potential ($\Delta\psi m$)-sensing dye $DiOC_6(3)$ for the evaluation of cell death–associated parameters by cytofluorometry. White and black columns illustrate the percentage of dying ($PI^- DiOC_6(3)^{low}$) and dead (PI^+) cells. Data are reported as means ± SEM ($n \geq 3$). *$p < 0.001$ (Mann–Whitney test), as compared with diploid cells subjected to the same treatment condition. #$p < 0.001$ (Mann–Whitney test), as compared to cells with the same ploidy status subjected to the same treatment condition but in absence of paclitaxel. Diploid, D; tetraploid, T.

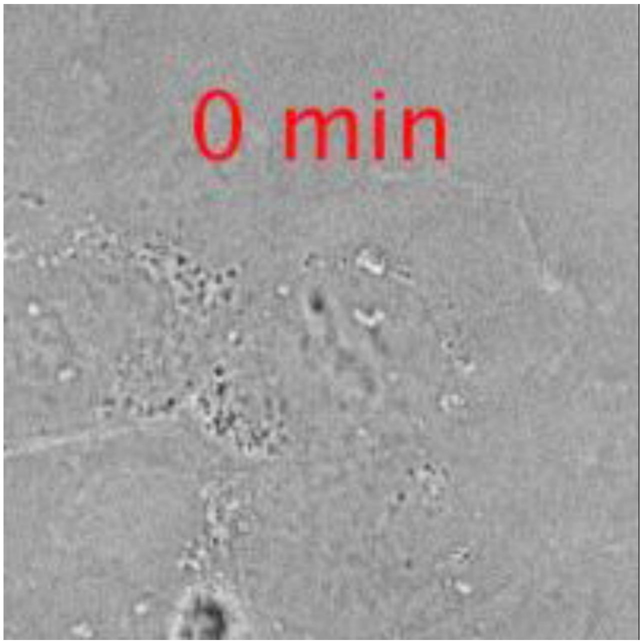

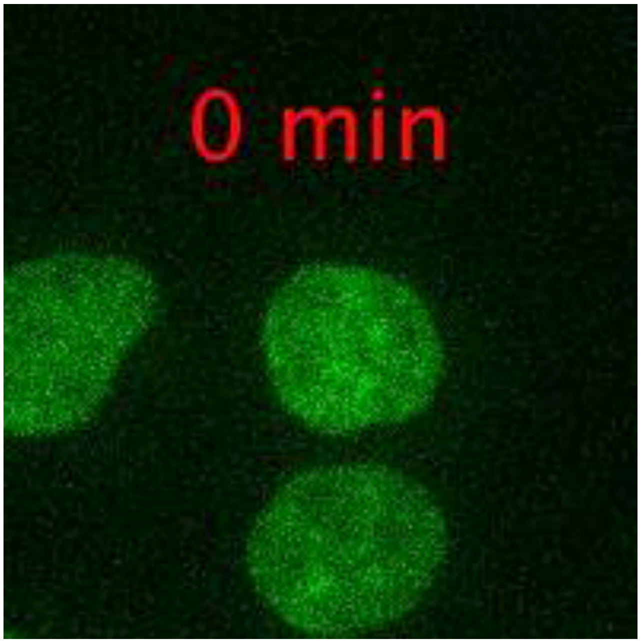

Supplementary Movies S1 and S2: Analysis of mitosis execution of tetraploid tumor cells in control conditions. Tetraploid human colon carcinoma HCT 116 cells stably expressing a green fluorescent protein tagged histone 2B variant (H2B-GFP) were transfected with an unrelated small interfering (si) RNA and monitored by videomicroscopy every 5 minutes to follow mitosis progression and analyze mitosis length. Supplementary Movie 1, bright-field; Supplementary Movie 2, green channel. Representative snapshots can be found in **Figure 4**.

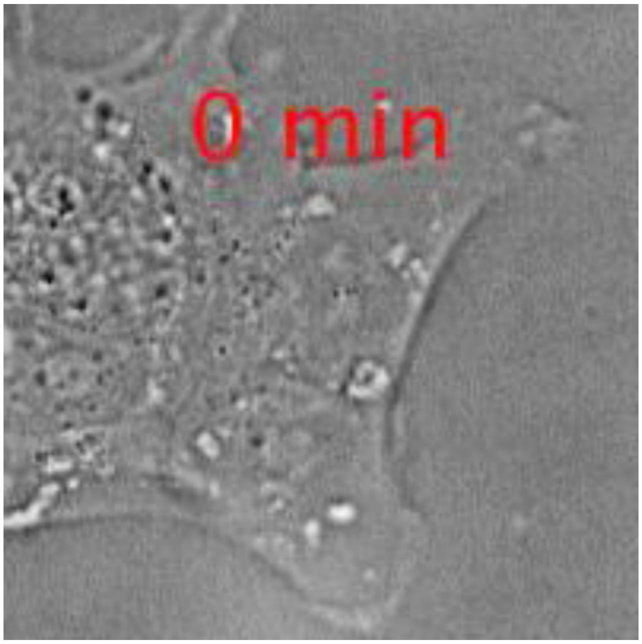

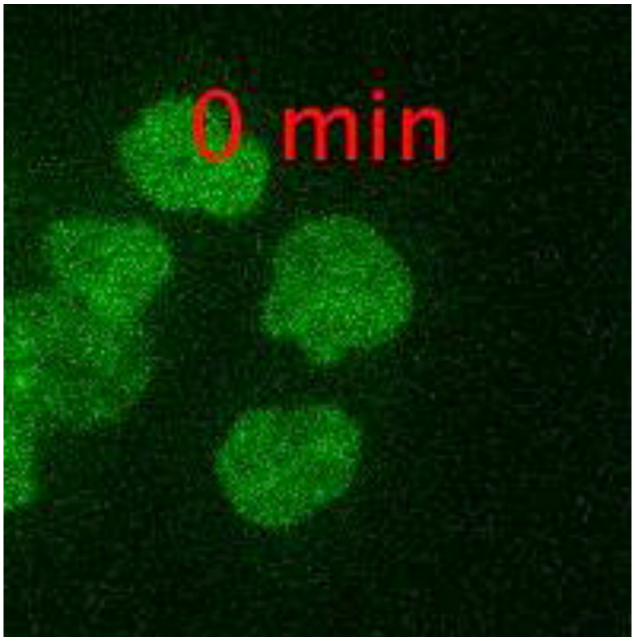

Supplementary Movies S3 and S4: Analysis of mitosis execution of tetraploid tumor cells depleted of MPS1. Tetraploid human colon carcinoma HCT 116 cells stably expressing a green fluorescent protein tagged histone 2B variant (H2B-GFP) were transfected with a specific small interfering (si) RNA directed against MPS1 and monitored by videomicroscopy every 5 minutes to follow mitosis progression and analyze mitosis length. Supplementary Movie 3, bright-field; Supplementary Movie 4, green channel. Representative snapshots can be found in **Figure 4.**

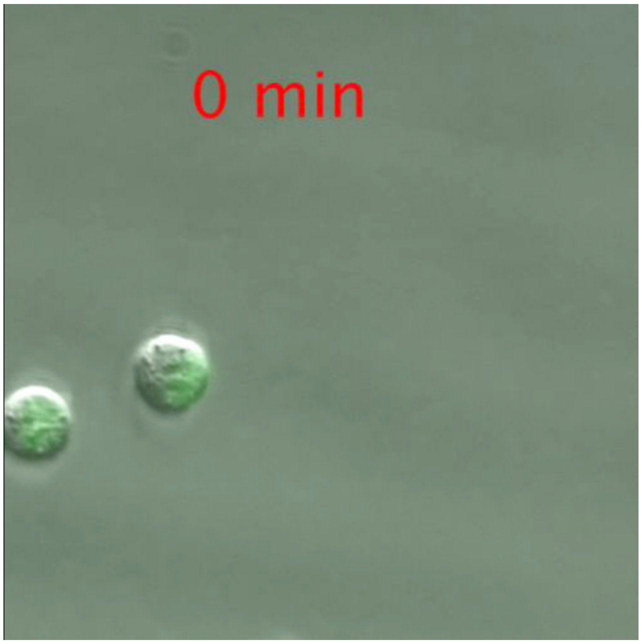

Supplementary Movie S5: Fate of tetraploid tumor cells in control condition. Tetraploid human colon carcinoma HCT 116 cells stably expressing a green fluorescent protein tagged histone 2B variant (H2B-GFP) were transfected with an unrelated small interfering (si) RNA and monitored by fluorescence videomicroscopy for 4380 min. Representative snapshots can be found in **Figure 5A**.

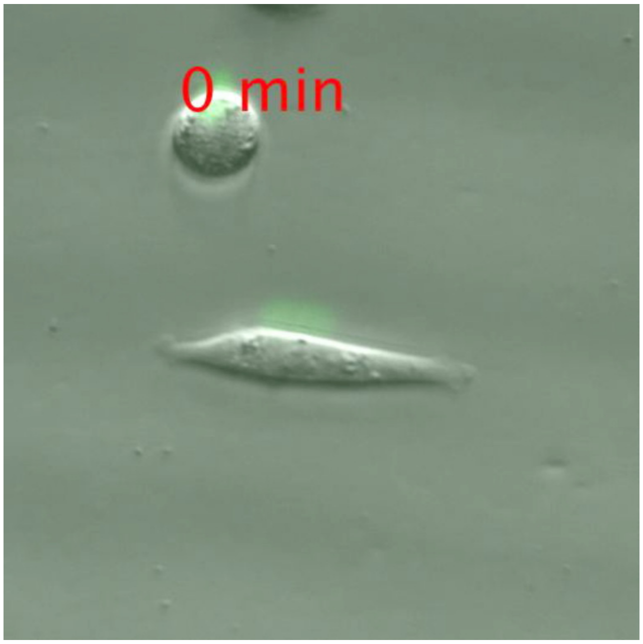

Supplementary Movie S6: Fate of tetraploid tumor cells depleted of MPS1. Tetraploid human colon carcinoma HCT 116 cells stably expressing a green fluorescent protein tagged histone 2B variant (H2B-GFP) were transfected with a specific small interfering (si) RNA directed against MPS1 and monitored by fluorescence videomicroscopy for 4380 min. This video shows one cell (located on the center of the view field) that undergoes an accelerated mitosis generating two daughter cells, both of which proliferate and enter an abortive mitosis generating one single polyploid (presumably octaploid) cell by cytokinesis failure and. Thereafter, one of these octaploid succumbs by apoptosis, while the other one undergoes a second abortive mitosis leading to a hyperploid (presumably decahexaploid) cell. Representative snapshots can be found in **Figure 5A**.
